# Hyperbaric oxygen therapy in the ATLS/ACLS resuscitative management of acutely ill or severely injured patients with severe anemia: a review

**DOI:** 10.3389/fmed.2024.1408816

**Published:** 2024-10-08

**Authors:** Keith W. Van Meter

**Affiliations:** Section of Emergency Medicine, Department of Medicine, LSU School of Medicine, New Orleans, LA, United States

**Keywords:** anemia, normobaric oxygen, hyperbaric oxygen, adenosine triphosphate, advanced cardiac life support, advanced trauma life support, oxygen debt, normobaric oxygen paradox

## Abstract

For short periods, even without the presence of red blood cells, hyperbaric oxygen can safely allow plasma to meet the oxygen delivery requirements of a human at rest. By this means, hyperbaric oxygen, in special instances, may be used as a bridge to lessen blood transfusion requirements. Hyperbaric oxygen, applied intermittently, can readily avert oxygen toxicity while meeting the body's oxygen requirements. In acute injury or illness, accumulated oxygen debt is shadowed by adenosine triphosphate debt. Hyperbaric oxygen efficiently provides superior diffusion distances of oxygen in tissue compared to those provided by breathing normobaric oxygen. Intermittent application of hyperbaric oxygen can resupply adenosine triphosphate for energy for gene expression and reparative and anti-inflammatory cellular function. This advantageous effect is termed the hyperbaric oxygen paradox. Similarly, the normobaric oxygen paradox has been used to elicit erythropoietin expression. Referfusion injury after an ischemic insult can be ameliorated by hyperbaric oxygen administration. Oxygen toxicity can be averted by short hyperbaric oxygen exposure times with air breaks during treatments and also by lengthening the time between hyperbaric oxygen sessions as the treatment advances. Hyperbaric chambers can be assembled to provide everything available to a patient in modern-day intensive care units. The complication rate of hyperbaric oxygen therapy is very low. Accordingly, hyperbaric oxygen, when safely available in hospital settings, should be considered as an adjunct for the management of critically injured or ill patients with disabling anemia.

## Preclinical introduction

The clinical use of hyperbaric oxygen therapy (HBOT) to address the absence of sufficient hemoglobin levels began with the work of Dutch surgeon, I. Boerema, in the late 1950s. He rapidly exsanguinated swine to hemoglobin levels as low as 1 g per deciliter and then resuscitated them by intravenous volume repletion with a Ringer's lactate–dextran 6%–dextrose water 5% solution. Next, he pressurized the unconscious, collapsed but still breathing, swine to three atmospheres of pressure in a hyperbaric chamber and made them breathe 100% oxygen. At three atmospheres of pressure, inhaled oxygen of 100% provided a surface equivalent fraction of inhaled oxygen of 300% (SEFIO_2_ 300%). He kept the swine at three atmospheres of pressure for 15 min and then re-transfused them with their shed blood and depressurized the chamber to the surface, whereupon the swine walked off unimpaired. He published these results in an article entitled “Life Without Blood” (1).

These results were replicated in a laboratory in the United States in 2010 at the LSU Health Sciences Center in New Orleans in an Institutional Animal Care Utilization Committee (IACUC)-approved pilot study. An acutely anesthetized, exsanguinated swine was monitored by a polarographic oxygen tension probe through a cranial burr hole (2). The swine, breathing normobaric room air, had a baseline brain tissue pO_2_ level of 30 mmHg. After a rapid exsanguination involving the removal of 40% of the blood volume, the swine's brain tissue pO_2_ dropped to 0 mmHg even while the swine was being ventilated with normobaric 100% oxygen. For volume replacement, the swine received intravenous Ringers' D5W solution. Next, the animal was pressurized inside a hyperbaric chamber while being kept on 100% oxygen inhalation at three atmospheres of pressure. At this pressure, the oxygen inhalation provided SEFIO_2_ of 300% oxygen. The brain tissue pO_2_ rose back to 30 mmHg, and the animal remained pressurized for 50 min. Before ascent to the surface, the swine was transfused with its shed blood. Upon reaching the surface at ambient pressure, the animal was recovered from anesthesia, and monitoring access catheters were removed. The swine walked off unimpaired and was returned to a rescue ranch for a long life (3). Table 1 shows a summary of published animal experiments investigating the use of HBOT in severe anemia. The tabular summary includes a thumbnail of evidence-based analysis using three different criteria (AHA/NCI-PDQ/BMJ) (4).

**Table 1:** Summary of published animal experiments investigating the use of hyperbaric oxygen therapy in severe anemia.

	**Date**	**References**	**Animal species**	**Study groups**	**Hemorrhagic insult**	**Survival rates**	**Thumbnail evidence-based analysis**			
1.	1943	Frank et al. (5)	Canine	1. Paper relied-on non-HBO2 controls from results of independent authors in the same model 2. Controlled study of 3 HBO2 0.3 MPa 150-180 min treatment group (*n* = 18)	Wiggers and Werle (6) “hypo-MAP” model for all animals	Survival at 4$\frac{1}{2}$ h post-hem: 1. non-HBO2 group (NBA or NBO2) = 0% 2. HBO2 groups = 20%	AHA		NCI-PDQ	BMJ evidence
							Level	Class	NA	NA
							6B	Indeter minate		
2.	1959	Burnet et al. (7)	Rat	Controlled study: 1. NBA/120 min group (*n* = 25) 2. HBO2 0.2 Mpa/120 min group (*n* = 25)	Intravascular hemolysis induced by 1 ml/100 g IM glycerol for all animals	Survival at 2 h. post insult, post-hem: 1. non-HBO2 group (NBA) = 20% 2. HBO2 group = 96%	AHA		NCI-PDQ	BMJ evidence
							Level	Class	NA	NA
							6A	II.b.		
3.	1959	Boerema et al. (1, 8)	Porcine	Controlled study: 1. HBO2 0.3 MPa/∴ι 75 min group (*n* = 3) HBO2 0.3 MPa with 30°C core temp/∴ι 75 min group (*n* = 20) NBA group (*n* = ?)	All animals were subjected to variable volume bleed which produced Hgb level of 0.4-0.6 g/dL	Survival at 45 min post-hem: HBO2 group = 100% HBO2 + hypothermic group = 50% NBA group = 0/2	AHA		NCI-PDQ	BMJ evidence
							Level	Class	NA	NA
							6B	II.b.		
4.	1962	Attar et al. (9)	Canine	Controlled study: 1. NBA group (*n* = 30) 2. HBO2 0.3 MPa/90 minutes group (*n* = 25)	Wiggers and Werle (6) “hypo-MAP” model for all animals	Survival at 48 h post-hem: NBA group = 17% HBO2 group = 74%	AHA		NCI-PDQ	BMJ evidence
							Level	Class	NA	NA
							6B	II.b.		
5.	1963	Cowley et al. (10)	Canine	Controlled study: 1. NBA with hem group (*n* = 30) 2. HBO2 with hem 0.3 MPa/150 min group (*n* = 19) 3. HBO2 without hem 0.3 MPa/150 min group (*n* = 13)	Wiggers and Werle (6) “hypo-MAP” model for all animals	Survival at 48 h post-hem: 1. NBA with hem group = 17% 2. HBO2 with hem group = 74% 3. HBO2 without hem group = 100%	AHA		NCI-PDQ	BMJ evidence
							Level	Class	NA	NA
							6A	II.b.		
6.	1964	Blair et al. (11)	Canine	Controlled study: NBA group (*n* = 23) HBO2 0.3 MPa/120 min group (*n* = 19)	Wiggers and Werle (6) “hypo-MAP” model for all animals	“Long-term” survival post-hem: NBA group = 17% HBO2 group = 74%	AHA		NCI-PDQ	BMJ evidence
							Level	Class	NA	NA
							6A	II.b.		
7.	1965	Clark and Young (12)	Canine	Controlled study: NBA group (*n* = 8) HBO2 0.2 MPa/150 min group (*n* = 5) NBA + IV bicarb group (*n* = 6)	Wiggers and Werle (6) “hypo-MAP” model for all animals	Survival at 18 h post-hem: NBA group = 75% HBO2 group = 100%	AHA		NCI-PDQ	BMJ evidence
							Level	Class	NA	NA
							6A	II.b.		
8.	1965	Cowley et al. (13)	Canine	Controlled study: 1. NBA group (*n* = 23) 2. HBO2 0.3 MPa/120 min group (*n* = 19)	Wiggers and Werle (6) “hypo-MAP” model for all animals	Survival at 48 h post-hem: 1. NBA group = 22% 2. HBO2 group = 74%	AHA		NCI-PDQ	BMJ evidence
							Level	Class	NA	NA
							6A	II.b.		
9.	1965	Elliot and Paton (14)	Canine	Controlled study: 1. NBA group (*n* = 10) 2. NBO2 group (*n* = 10) 3. NBO2 with ventilator group (*n* = 10) 4. HBO2 0.28 MPa/100 min group (*n* = 11)	Wiggers and Werle (6) “hypo-MAP” model for all animals	Survival at 72 h post-hem: 1. NBA group = 10% 2. NBO2 group = 50% 3. NBO2 with ventilator group = 50% 4. HBO2 group = 73%	AHA		NCI-PDQ	BMJ evidence
							Level	Class	NA	NA
							6A	II.b.		
10.	1965	Attar et al. (15)	Canine	Controlled study: Group I: 1. NBA/150 min subgroup (*n* = 25) 2. HBO2 0.3 Mpa/150 min subgroup (*n* = 29) Group II: Subgroup A 3. NBA/105 min group (*n* = 30) 4. HBO2 0.3 Mpa/105 min group (*n* = 22) Subgroup B 5. NBA/120 min group (*n* = 17) 6. NBO2/120 min group (*n* = 25) 7. HBO2/120 min group (*n* = 23) Subgroup C 8. NBA/150 min group (*n* = 30) 9. HBO2 0.3 MPa/ 0 min group (*n* = 4) Subgroup D 10. NBA/240 min group (*n* = 20) (*n* = ?) 11. HBO2 0.3 MPa/240 min group (*n* = 24) Group III: 12. HBO2 0.3 MPa/120 min started 30 min post-hem group (*n* = ?) 13. HBO2 0.3 MPa/120 min started 150 min post-hem group (*n* = ?) Group IV: 14. HBO2 0.2 MPa/120 min group (*n* = 11) 15. HBO2 0.2 MPa/150 min group (*n* = ?) 16. HBO2 0.3 MPa/120 min (*n* = 23) see above 17. HBO2 0.3 MPa/130 min (*n* = 4)see above	Wiggers and Werle (6) “hypo-MAP” model for all animals	Survival at 72 h post-hem: 1. NBA group = 20% 2. HBO2 group = 41% 3. NBA group = 66% 4. HBO2 group = 47% 5. NBA group = 29% 6. NBO2 group = 20% 7. HBO2 group = 72% 8. NBA group = 17% 9. HBO2 group = 50% 10. NBA group = 50% 11. HBO2 group = 48% 12. HBO2 30 min post- hem = 74% 13. HBO2 150 min post-hem 50% 14. HBO2 0.2 MPa/120 min = 82% (*n* = ?) 15. HBO2 0.2 MPa/150 min = 30% 16. HBO2 0.3 MPa/120 min = 72% 17. HBO2 0.3 MPa/150 min = 50%	AHA		NCI-PDQ	BMJ evidence
							Level	Class	NA	NA
							6A	II.b.		
11.	1965	Jacobsonet al. (16, 17)	Rabbit	Controlled study: 1. NBA group (*n* = 10) 2. NBO2 group (*n* = 10) 3. HBO2 0.2 MPa/12 h (*n* = 10)	Wiggers and Werle (6) “hypo-MAP” model for all animals	Survival at 48 h post-hem: 1. NBA group = 0% 2. NBO2 group = 10% 3. HBO2 group = 10%	AHA		NCI-PDQ	BMJ evidence
							Level	Class	NA	NA
							6A	II.b.		
12.	1965	Whalen et al. (18)	Canine	Controlled study: 1. NBA group (*n* = 5) 2. NBO2 group (*n* = 5) 3. HBO2 0.35 MPa (*n* = 5) 4. HBO2 0.35 MPa	Complete replacement of blood volume of group 4 animals with dextran 6%/dextrose, 5%/RL solution to produce a Hct of 0.5%	All groups 100% survival, but group 4 had increased cardiac output and decreased peripheral vascular resistance	AHA		NCI-PDQ	BMJ evidence
							Level	Class	NA	NA
							6B	Indeter minate		
13.	1965	Navarro and Ferguson (19)	Canine	Controlled study: 1. NBA/120 min dextran group (*n* = 15) 2. NBA/120 min dextrose group (*n* = 15) 3. HBO2 0.35 MPa/120 min dextran group (*n* = 15) 4. HBO2 0.35 MPa/120 min dextrose group	Wiggers and Werle (6) “hypo-MAP” model for all animals	Survival at 48 h post-hem after administration of exp: 1. NBA dextran group = 26% 2. NBA dextrose group = 6.6% HBO2 dextran group = 60% 3. HBO2 dextrose group = 60%	AHA		NCI-PDQ	BMJ evidence
							Level	Class	NA	NA
							6A	II.b.		
14.	1969	Necas and Neuwirt (20)	Rat	Controlled study: 1a. NBA group (*n* = 5) 2a. HBO2 0.3 MPa/360-420 min group (*n* = 3) 3a. HBO2 0.2 MPa/360-420 min group (*n* = 2) 1b. NBA group (*n* = 3) 2b. HBO2 0.3 MPa/360-420 min group (*n* = 4) 3b. NBA group (*n* = 13) 4b. HBO2 0.3 MPa/360-420 min group (*n* = 9)	Group a: Hemorrhage to Hct of 25% Group b: Hemorrhage to Hct of 10%	Survival at h: 1a. NBA with Hct 25% group = 60% 2a. HBO_2_ with Hct 25% group = 100% 3a. HBO_2_ with Hct 25% group = 100% 1b. NBA with Hct 10% = 0% 2b. HBO_2_ with Hct 10% = 100% 3b. NBA with Hct 10% group = 0% 4b. HBO2 with Hct 10% = 100%	AHA		NCI-PDQ	BMJ evidence
							Level	Class	NA	NA
							6A	II.b.		
15.	1970	Doi and Onji (21)	Canine	Controlled study: 1. NBA/90 min (∴ι120 min) group (*n* = 7) 2. HBO2 0.2 MB/90 min (∴ι 120 min) group (*n* = 7)	Wiggers and Werle (6) “hypo-MAP” model for all animals	Survival at 4$\frac{1}{2}$ h post-hem: 1. NBA group = 0% 2. HBO2 group = 100%	AHA		NCI-PDQ	BMJ evidence
							Level	Class	NA	NA
							6A	II.b.		
16.	1970	Oda and Takeori (22)	Canine	Controlled study: 1. NBA NS/5% dextran 40/80 min (*n* = 5) 2. HBO2 0.3 MPa/60 min NS/3% dextran 40 group (*n* = 5) 3. NBA NS/5% dextran 200 group (*n* = 5) 4. HBO2 0.3 MPa/60 min NS/6% dextran 200 group (*n* = 5)	25 ml/kg shed blood with exchange of NS designated exchange followed by continued bleed to produce a Hct of 18%	Survival rates: 1. NBA dextran 40 group = 100% 2. HBO2 dextran 40 group = 100% 3. NBA dextran 200 group = 100% 4. HBO2 dextran 200 group = 100%	AHA		NCI-PDQ	BMJ evidence
							Level	Class	NA	NA
							6A	Indeter minate		
17.	1974	Trytyshnkov (23)	Rat	Controlled study: 1. NBA no hem group 2. NBA with hem group 3. HBO2 0.2 MPa/60 min no hem group 4. immediate HBO2 0.2 MPa/60 min post-hem group 5. delayed HBO2 0.2 MPa/60 min post-hem group (total *n* = 179)	3% body weight hemorrhage by jugular blood draw over 30 min	Survival rates: 1. NBA group = 100% 2. NBA with hem group = 0% 3. HBO2 no hem group = 100% 4. immediate HBO2 post-hem group = 100% 5. delayed HBO2 post- hem group = 0%	AHA		NCI-PDQ	BMJ evidence
							Level	Class	NA	NA
							6B	II.b.		
18.	1975	Norman (24)		Controlled study:			AHA		NCI-PDQ	BMJ evidence
							Level	Class	NA	NA
							6A	II.b.		
19.	1976	Barkova and Petrov (25)	Rat	Non-controlled study: 1. NBA group (*n* = 60) 2. HBO2 0.2 MPa/40 min group (*n* = 60)	2.8% of body weight blood loss over 30 min	Survival rate: 1. NBA group = 0% 2. HBO2 group = 100%	AHA		NCI-PDQ	BMJ evidence
							Level	Class	NA	NA
							6B	II.b.		
20.	1977	Luonov and Takovlev (26)	Cat	Controlled study: 1. NBA/60 min group (*n* = ”?”) 2. HBO2 0.3 MPa/60 min group (*n* = ”?”)	Wiggers and Werle (6) “hypo-MAP” model for all animals	Survival rate: 1. NBA group = increase in brain ammonia 2. HBO2 group = no increase in brain ammonia	AHA		NCI-PDQ	BMJ evidence
							Level	Class	NA	NA
							6B	Indeter minate		
21.	1983-84	Gross et al. (27–29)	Canine	Controlled study: 1. NBA 6% dextran 40 group (*n* = 6) 2. NBA RL group (*n* = 6) 3. NBA 10% dextrose group (*n* = 6) 4. NBA 6% dextran 70 group (*n* = 6) 5. HBO2 0.28 MPa/93-118 min 6% dextran-40 group (*n* = 6) 6. HBO2 0.28 MPa/93-118 min RL group (*n* = 6) 7. HBO2 0.28 MPa/93-118 min 10% dextrose (*n* = 6) 8. HBO2 9.28 MPa/93-118 min 6% dextran-70 group (*n* = 6) 9. HBA 0.6 MPa 6% dextran-40 (*n* = 6) 10. HBA 0.6 RL group (*n* = 6) 11. HBA 0.6 MPa 10% dextran group (*n* = 6) 12. HBO2 0.6 MPa 6% dextran-70 group (*n* = 6)	Wigger and Werle (6) “hypo-MAP” model for all animals	Survival post-hem: 1. NBA 6% dextran-40 group = 100% 2. NBA RL group = 100% 3. NBA 10% dextrose group = 100% 4. NBA 6% dextran 70 group = 100% 5. HBO2 6% dextran- 40 group = 100% 6. HBO2 RL group = 100% 7. HBO2 10% dextrose group = 100% 8. HBO2 6% dextran-70 group = 100% 9. HBA 6% dextran-40 grou p = 100% 10. HBA RL group = 100% 11. HBA 10% dextrose group = 100% 12. HBA 6% dextran-70 group = 100%	AHA		NCI-PDQ	BMJ evidence
							Level	Class	NA	NA
							6A	Indeter minate		
22.	1991	Bitterman et al. (30)	Rat	Controlled study: 1. NBA sham group (*n* = 6) 2. NBA + hem group (*n* = 10) 3. NBO2/90 min + hem group (*n* = 10) 4. HB nitrox (7/93) 0.3 MPa/190 min + hem group (*n* = 8) 5. HBO2 0.3 MPa/90 min sham group (*n* = 6) 6. HBO2 0.3 MPa/90 min + hem group (*n* = 10)	Hemorrhage within 90 min of 3.2 ml all animals so designated	Survival post-hem: MAP > 40 mmHg for 220 min: 1. NBA sham group = 100% 2. NBA + hem group = 10% 3. NBO2 + hem group = 50% 4. HB nitrox + hem group = 0% HBO2 sham group = 100% 5.HBO2 + hem group = 100%	AHA		NCI-PDQ	BMJ evidence
							Level	Class	NA	NA
							6A	II.b.		
23.	1992	Wen-Ren (31)	Canine	Controlled study: 1. NBA/95 min group (*n* = 6) 2. HBO2 0.3 MPa/95 min group (*n* = 6)	Hemorrhage to 60 ml/kg	Survival rate: 1. NBA group = 0% 2. HBO2 group = 100%	AHA		NCI-PDQ	BMJ evidence
							Level	Class	NA	NA
							6A	II.b.		
24.	1992	Marzella et al. (32)	Rat	Controlled study: 1. NBA hem with 90 min monitoring group (*n* = ”?”) 2. HBO2 hem 15 min then 0.2 MPa/75 min with monitoring group (*n* = ”?”)	Hemorrhage to 15 ml/kg	Survival rates not provided for groups 1. NBA group: BP decreased 25%, CO decreased 25% 2. HBO2 group: BP increased 10%, CO decreased 25%	AHA		NCI-PDQ	BMJ evidence
							Level	Class	NA	NA
							6B	Indeter minate		
25.	1995	Adir et al. (33)	Rat	Controlled study: 1. NBA no hem group (*n* = 11) 2. NBA + hem group (*n* = 10) 3. NBO2 + hem group (*n* = 10) 4. HBO2 0.3 MPa/90 min no hem group (*n* = 7) 5. HBO2 0.3 MPa/90 min + hem group ( *n* = 10)	Hemorrhage 3.2 ml/100 g over 120 min for all animals so designated	Survival at 24 hour/7 day post-hem: 1. NBA no hem group = 100%/45% 2. HBA hem group = 70%/10% 3. NBO2 hem group = 90%/70% 4.HBO2 no hem group = 100%/55% 5. HBO2 hem group = 90%/10%	AHA		NCI-PDQ	BMJ evidence
							Level	Class	NA	NA
							6A	Indeter minate		
26.	2000	Yamashita and Yamashita (34)	Rat	Controlled study: 1. NBA + hem group (*n* = 15) 2. HBO2 0.3 MPa/60 min with 30 min decompression + hem group (*n* = 10) 3. HBO2 0.3 MPa/60 min with 30 min decompression no hem group (*n* = 10)	Hemorrhage of 40 ml/kg over 1 h	Survival at 24 h post-hem: 1. NBA + hem group = 40% 2. HBO2 + hem group = 83% 3. HBO2 no hem group = 100%	AHA		NCI-PDQ	BMJ
							Level	Class	NA	NA
							6A	IIb		

## The clinical use of hyperbaric oxygen

Hyperbaric oxygen (HBO) may be used as a bridging therapy in the Advanced Trauma Life Support (ATLS) and Advanced Cardiac Life Support (ACLS) resuscitation of a precariously anemic patient to prevent multiunit transfusion until damage control surgical efforts can be implemented. The initial damage control surgery aims at preventing continued blood loss to allow the patient to retain transfused blood (35).

Likewise, HBO may be used as a bridging therapy for patients who refuse blood transfusions due to religious or philosophical reasons. Tincture of time could then allow the provision of hematinic nutrients and pharmaceuticals to support hematopoiesis to endogenously provide red blood cell replacement (36). If hemoglobin's ability to transport oxygen by carbon monoxide, cyanide, or hydrogen sulfide is impaired, HBO can be used acutely to treat these conditions to assist in patient recovery from the chemical hypoxia imposed by the poisoning (37–41).

In yet another clinical instance, HBO may be used if an anticipated complication of a blood transfusion precludes further transfusion (42):

1. Blood group incompatibility.
2. Febrile non-hemolytic transfusion reaction (FNHTR).
3. Both delayed amnestic and primary hemolytic anemia.
4. Allergy from urticaria to anaphylaxis.
5. Transfusion-associated graft-versus-host disease (TAGVHD).
6. Acute radiation-induced anemia in disasters with a supply shortage.
7. Transfusion-transmitted infections (TTI).
8. Both red blood cell and human leukocyte antigen (HLA) allosensitization.
9. Confounding severe congestive heart failure with profound anemia until stabilization, providing the safety of transfusion.
10. Stacking hemosiderosis from multiple transfusions by lessening the number of transfusions.
11. The prevention of long-term transfusion immunomodulation by lessening the number of transfusions.
12. The prevention of short-term induction of multiorgan failure by red blood cell-associated lipids and cytokines.
13. Transfusion-related lung injury (TRALI).

HBOT has been documented to ameliorate the adult respiratory syndrome induced by trauma or infection in severely anemic patients (43–46). This effect of HBOT may also be found to be an additional advantage of HBO as a bridging treatment until safe transfusions are possible in a patient with TRALI or acute respiratory distress (ARDS) in SARS-CoV2 patients with severe anemia (47). Research into this area is necessary. A randomized, controlled study has been published evidencing the use of HBOT to perform this (44). Table 2 shows human case studies and series for use in the treatment of severe anemia. The tabular summary includes a thumbnail, evidence-based analysis of the published papers using three different criteria (AHA/NCI-PDQ/BMJ) (4). More recently, a randomized, controlled trial of HBOT used in severe anemia has been published (57).

**Table 2:** Human case reports and series for use of hyperbaric oxygen therapy in treatment of severe anemia.

	**Date**	**References**	**Pt age/gender**	**Quantification of hemorrhagic insult**	**Adjunctive transfusion**	**Adjunctive hematinics and HBO2**	**Survival**	**Thumbnail evidence-based analysis**			
1.	1969	Ledingham (48)	40 y/female	Admission Hgb = 1.5 g/dL Admission BP = 65/? Admission sensorium = AMS	Yes (patient was transfused after stabilization by completed HBO2)	B12 folic acid, ascorbic acid HBO2 0.2 Mpa/5 hr + (at depth the pt would seize at first when oxygen mask was removed)	Yes	AHA		NCI-PDQ	BMJ evidence
								Level	Class	3.iii.	Likely to be beneficial
								5	Indeter minate		
2.	1969	Amonic et al. (49)	26 y/male (JW)	S/P resuscitation of leiomyoma to resolve GI bleed Post-op Hct = 10% 3rd post-op day = CHF Serial HBO2 7th post-op day Hct = 12% 7th post-op week Hct = 42%	No	Hematinics – yes Serial 17 cycles of HBO2 0.2 MPa/160 min (at depth the pt initially seized when oxygen mask was removed)	Yes	AHA		NCI-PDQ	BMJ evidence
								Level	Class	3.iii.	Likely to be beneficial
								5	Indeter minate		
3.	1974	Hart (50)	27 y/female (JW) 67 y/female (JW) 27 y/male (JW)	Perinatal pelvic hematoma and pulmonary embolism with Hgb 3.8 g/dL, congestive heart failure, AMS, and 88/40 BP Diverticulosis with rectal bleeding with Hgb 2.6 g/dL, AMS, and 90/70 BP MVA with liver laceration with Hgb 6.9 g/dL	No Yes (pt was transfused 2 units PRBC's on 4th hospital day after Continued bleeding) No	Iron dextran IM Serial HBO2 0.2 MPa/90 Iron dextran IM Serial HBO2 0.2 MPa/90 Iron dextran IM Serial HBO2 0.2 MPa/90	Yes Yes Yes	AHA		NCI-PDQ	BMJ evidence
								Level	Class	3.iii.	Likely to be beneficial
								5	Indeter minate		
4.	1974	Myking and Schreinen (51)	55 y /female	AIHA with HGB 4.6 g/dL Failed prednisone with Hgb falling to 3 g/dL with AMS Serial HBO2 x 5 days with Hgb 5 g/dL	No	Prednisone Serial HBO2 0.26 MPa/240 min QID tapered to HBO2 0.26 MPa/120 min BID to day 5 with D/C	Yes	AHA		NCI-PDQ	BMJ evidence
								Level	Class	3.iii.	Likely to be beneficial
								5	Indeter minate		
								Level	Class		
5.	1987	Hart et al. (52)	20 females (JW) 6 males (JW) (subgroup analysis of those patients without AMS leaves 19 pts)	Mean Hct of all 26 patients = 13% (all with class IV hem)	No No	All had hematinics, vitamin B12, vitamin c, iron All patients averaged 9.6 HBO2 sessions 0.2 MPa/90 min	65% 83% 95%	AHA		NCI-PDQ	BMJ evidence
								Level	Class	3.iii.	Beneficial
								5	II.b.		
6.	1989	Myerstein et al. (53)	4 individual human blood samples were tested for levels of GSH, Hct/ free Hgb, MetHgb, and RBC volume	Study groups: Control RBCs, both fresh and stored samples Low GSH RBCs induced by diamide in both fresh and stored samples RBCs exposed to HBO2 0.3 MPa/120 min in both fresh and stored samples 4. Low GSH RBCs induced by diamide in both fresh and stored samples exposed to HBO2 0.3 MPa/120 min			No damage or abnormality induced by HBO2 over controls	AHA		NCI- PDQ	BMJ evidence
								Level	Class	NA	NA
								6	II.b.		
7.	1992	Young and Burns (54)					Yes	AHA		NCI- PDQ	BMJ evidence
								Level	Class	3.iii.	Likely to be beneficial
								5	II.b.		
8.	1999	McLoughlin et al. (55)	38 y/female	Antepartum hemorrhage with Hgb 2 g/dL 39 day post-bleed discharge Hgb 7.6 g/dL	No	Vitamin B12, EPO, folic acid, iron HBO2 0.3 MPa/90 min TID tapered to BID over 16 days (total 22 HBO2 sessions)	Yes	AHA		NCI- PDQ	BMJ evidence
								Level	Class	3.iii.	Likely to be beneficial
								5	II.b.		
9.	2002	Hart (56)	20 y/female (JW)	GSW to left chest with left lung and hemidiaphragm penetration with spleen, left kidney and spinal cord injury Post-op Hct 18 Post-op intestinal perforation Post-op day 28 Hct 22	No	EPO HBO2 0.2 MPa/90 min TID tapering to BID for a total of 28 dives	Yes	AHA		NCI- PDQ	BMJ evidence
								Level	Class	3.iii.	Likely to be beneficial
								5	II.b.		

**Figure 1:**
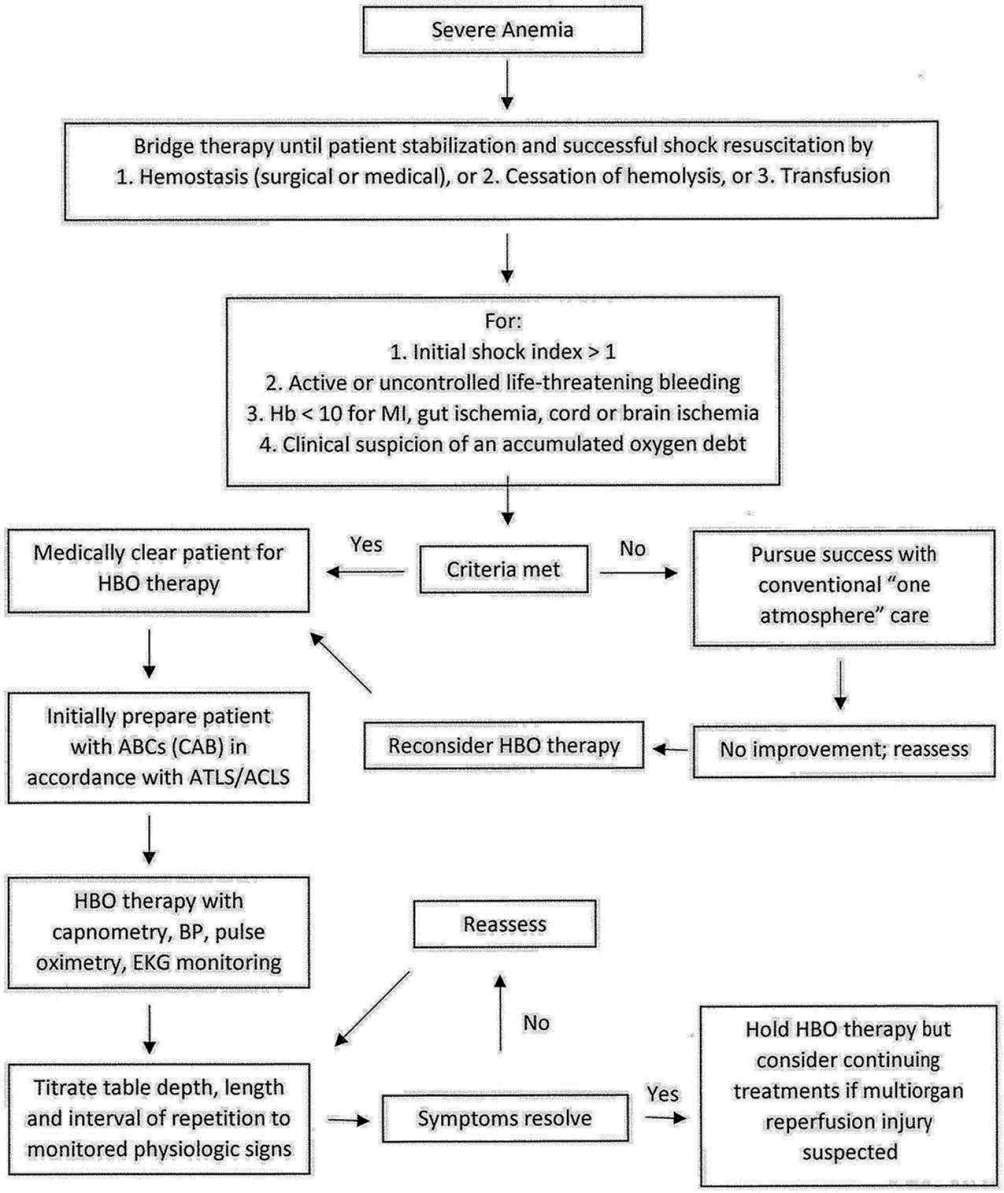
Flowchart of Severe Anemia.

When considering the use of HBOT in cases of severe anemia, the clinician should consult a hyperbaric physician specialist to determine whether HBOT would be helpful for the individual patient. Figure 1 demonstrates the treatment course recommended by the UHMS in their 2023 edition of the Hyperbaric Medicine Indications Manual. The patient would undergo HBOT at two–three ATA for 60–90 min with one–two intermittent 5-min air breaks (4).

## Discussion

The operational practicality of using HBO as a bridging therapy in a remote non-medical setting was reported in a case of severe exsanguination of a commercial diver while in saturation offshore in the Gulf of Mexico. The patient bled out hemoglobin of 2 g per deciliter acutely when his duodenal artery was eroded by a duodenal ulcer. The requisite decompression to the surface required 3 days. During his recompression, he was kept alive without transfusion by Ringers' D5 solution administered by hypodermoclysis and intermittent HBO breathing periods. At the surface, he was then transfused with intravenous packed red blood cells (58). Many years before, three cases of patients with severe blood loss who each refused transfusion for religious belief were reported in the medical literature. The cases were successfully treated with intermittently administered HBO in the same way at a naval dock in a hyperbaric chamber (50).

High concentrations of continuously administered oxygen have been reported to be deleterious when used in patient's resuscitative management (59, 60). This observation has remained consistent regardless of whether the patients enrolled in clinical trials have had high, normal, or low hemoglobin levels, whether acute or chronic (61). How could HBO provided by ventilation with SEFIO_2_ of inhaled oxygen of 150%−300% not be deleterious? For one, the inhaled oxygen under these conditions is not continuous but is intermittent, with administered air breaks incorporated during HBOT sessions (62). Additionally, as the series of HBO treatment sessions progresses and the patient's condition improves, the patient becomes increasingly tolerant of the off-oxygen periods. This allows the HBOT to be spread out with longer periods between treatments (49). During the HBO breathing periods, enough oxygen is dissolved in plasma to allow plasma to deliver oxygen to tissue mitochondria to reduce the previously accumulating oxygen debt, which, in effect, is an adenosine triphosphate (ATP) debt (63–66).

One might say that HBOT, as bridge therapy, serves to resuscitate patients much like the bridging function of veno-venous extracorporeal membrane oxygenation (VV-ECMO) during resuscitative support of critically anemic patients with restrictions on red blood cell transfusion. In the instance of intermittent non-invasive HBOT, the patient can similarly be successfully supported (67). By simile, one might compare VV-ECMO to a continuous weld and short-interval intermittent HBOT to a spot weld (in effect, intermittent HBOT is VV-ECMO-like or “ECMoid” in function). Both therapeutic modalities attempt to hold the metabolic structure of the patient together. ECMO has up to a 30% serious adverse side effect incidence (68). Hyperbaric oxygen has, on average, one in 10,000 incidences of serious side effects including pneumothorax, oxygen toxicity seizure, fire or explosion, and arterial gas embolism (69–71). The ECMO hospital facility support fee is often US $50,000 per day (72) and a hospital-based HBOT series of 30 treatments includes a facility charge of US $7,500 (73, 74). In almost all instances, 30 HBOT treatments would be more than enough to bridge a patient through an anemic crisis. In the United States, the cost of a unit of packed red blood cells, along with its administration, is comparable to the cost of one HBOT treatment (4).

The tolerance to high-dose oxygen administration by intermittent application has been well-documented with oxygen administered at one atmosphere pressure as well as at increased atmospheric pressure (62, 75). There is more than just oxygen tolerance provided by the intermittency of use; this is the effect of intermittency itself. ATP resupply occurs when the mitochondrial intermembrane space minimally attains 1.5–2.0 mmHg of oxygen, which is a requisite for the unimpaired production of ATP by the mitochondrial respiratory chain of enzymes (76). In a severely anemic patient, equally important is the return of tissue hypoxia after the completion of an HBOT treatment. It is hypoxia that incites ATP-dependent reparative cytokine tissue release and antioxidant production. For the reparative and anti-inflammatory cytokines to work at cellular receptor sites, ATP is needed. The anteceding HBOT would have supplied the needed ATP for this to occur. The ensuing tissue hypoxia between treatments induces the following energy-dependent or ATP-dependent activity:

1. Antioxidant productions and functions to include catalase and peroxidase (77), glutathione (78), superoxide dismutase (79), and ATP itself as an antioxidant (80).
2. Support of genomic activity (81).
3. Support of epigenomic activity (82).
4. Support of proteomic activity (83) and protein folding (84).
5. Support of lipidomic activity (85).
6. Support of anti-inflammatory and reparative cytokines/chemokines (86).
7. Leukocyte function (87, 88).
8. Adaptive function in hypoxia: (erythropoietin) (89, 90) (heat shock protein) (91) (nitric oxide) (92) (hypoxia-inducible factor) (93).

**Figure 2:**
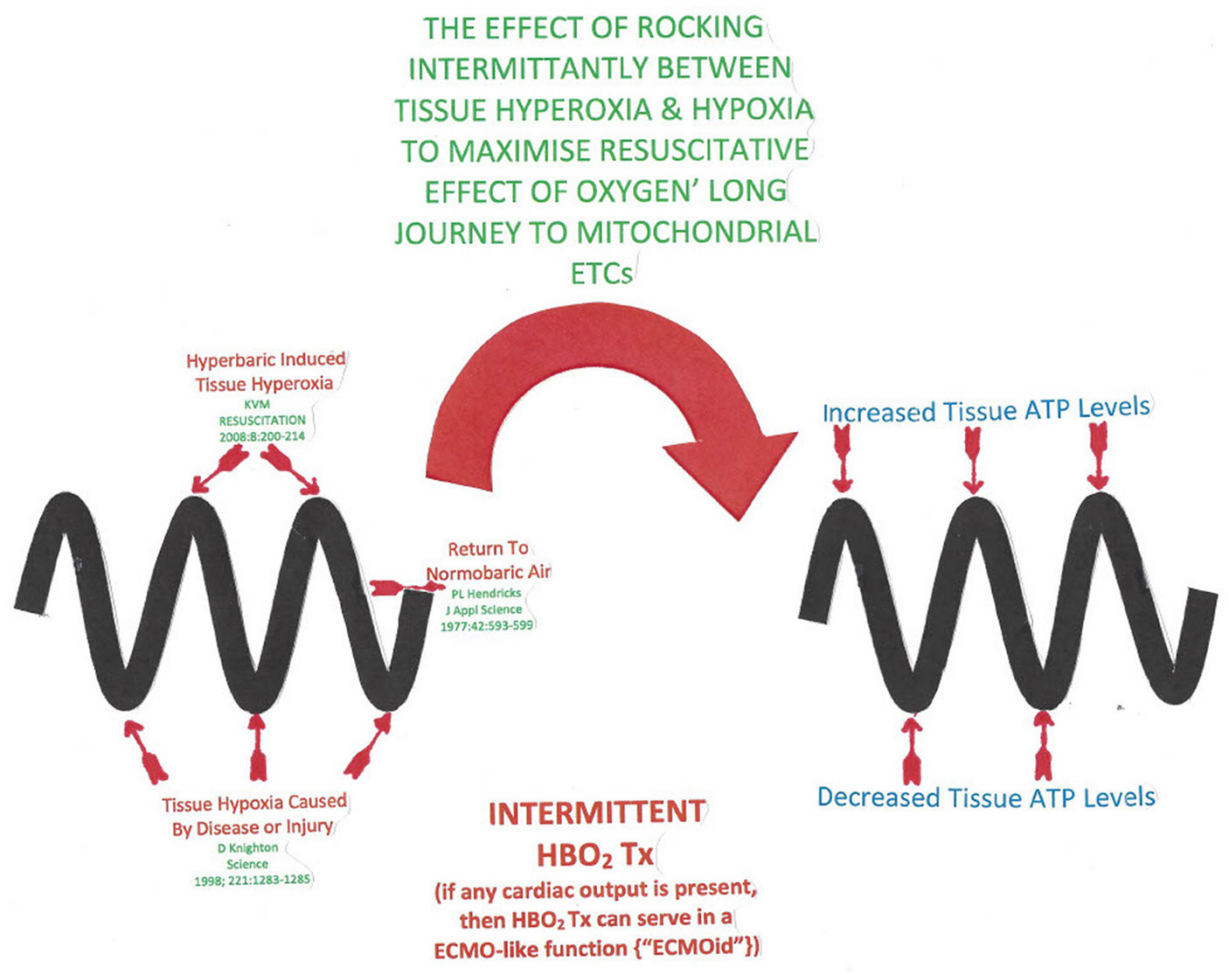
The sinusoidal horizontal timeline depicted by the thick wavy lines in the diagram represents an intermittent hyperbaric oxygen treatment course. The wave peaks represent a hyperbaric oxygen treatment producing ATP resupply (93). Post treatment when the tissue oxygen tension drops, energy requiring cytokine and antioxidant release occurs (85).

This oscillation between hyperoxia and hypoxia may be graphically depiected as a sinusoidal timeline by Figure 2 and is the crux of the oxygen paradox.

To provide ATP resupply in the instance of an acute hypoxic state, pulsed high-dose HBO inhalation can be used to diffuse minimally 1.5–2.0 mmHg of oxygen into the mitochondrial intermembrane space (94). At best, when a red blood cell gets to its destination in capillaries, it must offload a portion of its remaining oxygen content back into the plasma. As the patient inhales 100% oxygen at one atmosphere of pressure, the plasma can maximally contain only 2.3 volumes% of dissolved oxygen. In contrast, the patient in a hyperbaric chamber at three atmospheres of pressure would inhale a SEFIO_2_ of 300%, thereby delivering to the capillaries 6.6 volume% of dissolved oxygen with a five-fold diffusion distance outside of the capillary over that of a subject inhaling an FIO_2_ of 100% oxygen at one atmosphere of pressure (95–97). This concept was reported over 60 years ago by W. Brummelkamp when he reported that during an HBOT treatment, “drenching of the tissue with dissolved oxygen” occurred by way of immersing plasma with oxygen (98).

HBOT inhalation can only be accomplished safely when the entire patient is pressurized above ambient pressure in an enclosure (i.e., a hyperbaric chamber). The spectrum of potential treatment doses of oxygen using HBO pressure incorporates the pharmacologic effect of the gases at increased pressure and the physiologic effect of pressure itself (99).

A measure of the safety of HBO can best be described by Pascal's Law, where in a confined space, any contained fluid will transmit the pressure evenly throughout the fluid non-destructively. The human skin envelope contains the fluid of all the body's tissue (gas is not a problem in sinus spaces if vented by an open ostia and in the middle ear if vented by a patient's functioning Eustachian tube) (100). By virtue of the principle of Pascal's law, a patient may be ventilated without barotrauma by pressurized gas at the same pressure as that of hyperbaric chamber pressurization. Ventilators have been developed to do this safely, and chambers can be fitted with all the functions of critical care hospital units (101, 102).

Henry's Gas Law states that the concentration of a solute gas in a solution is directly proportional to the partial pressure of the gas over the solution. Inhalation of HBO at three atmospheres of pressure allows enough dissolved oxygen (6.6 volumes%) in plasma to supply the metabolic extraction rate of most of the tissue in a human body at rest (103).

The operational safety of hyperbaric medicine units has evolved through adherence to developing safety guidelines. This has allowed a remarkable safety record for hospital-based units for equipment, patients, and healthcare providers for both multiplace and monoplace chamber facilities (104, 105).

At the 21% oxygen content of air in one atmosphere, hemoglobin makes up for plasma's inability to deliver adequate oxygen to tissue. This is because a subject breathing air would have, at maximum, a 0.48 volume% of plasma dissolved oxygen, which clearly would not be enough to support human life (0.003 ml × 21% × 760 mmHg, where 0.003 ml is the amount at one atmosphere of oxygen dissolved in plasma for each mmHg of pressure, 21% is the oxygen content of air, and 760 mmHg is the pressure for each mmHg of pressure in the atmosphere at sea level) (106). Using the same equation for breathing 100% oxygen at one atmosphere, the maximum amount of dissolved oxygen in plasma would be 2.3 volume%. As mentioned, this would be far below the average oxygen extraction rate of most human tissue with the body at rest. To get around this problem, hemoglobin serves as a powerful gas clathrate, especially for oxygen. When a red blood cell picks up oxygen in the lung and discharges it in the periphery, the maximum oxygen conceivably dissolved in plasma would be 2.3 volume% at both ends of the line.

**Figure 3:**
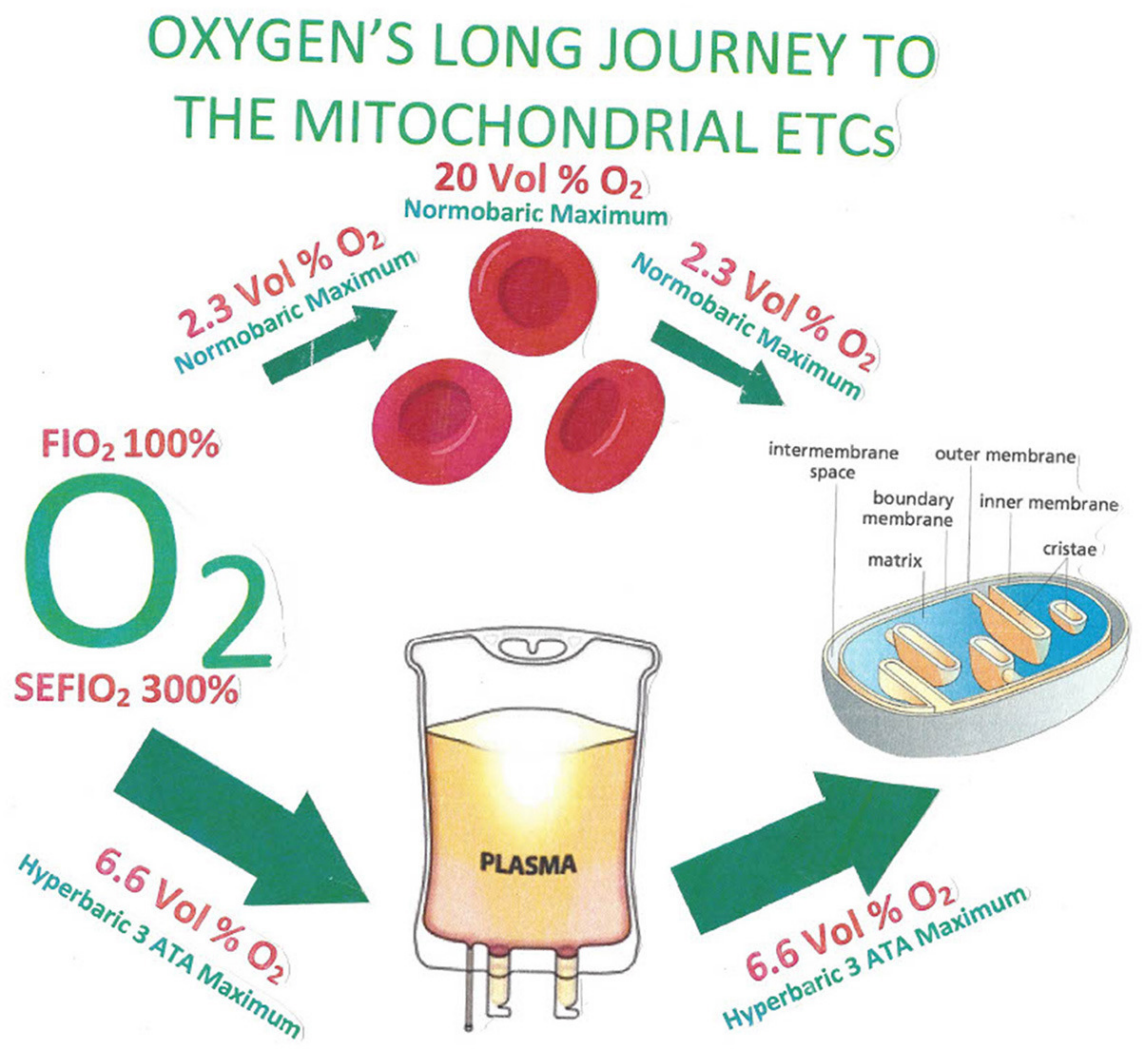
The most oxygen that can conceivably be dissolved in plasma by a subject who breathes 100% oxygen is 2.3 volume%, and the plasma level enters the red blood cells in the lung capillary, where the blood content can be boosted as high as 20 volume% by the presence of the gas-clathrate-like function of hemoglobin. When the red blood cell gets to its destination in a capillary of distant tissue, the highest possible concentration as the oxygen unloads from the red blood cell into the plasma possible at normobaric pressure is again at the very highest, 2.3 volume%. Under hyperbaric conditions at three atmospheres of pressure, the equivalent amount of oxygen possible in plasma during the circulatory route all the way to distal capillaries at the very highest would be 6.6 volume% (108).

**Figure 4:**
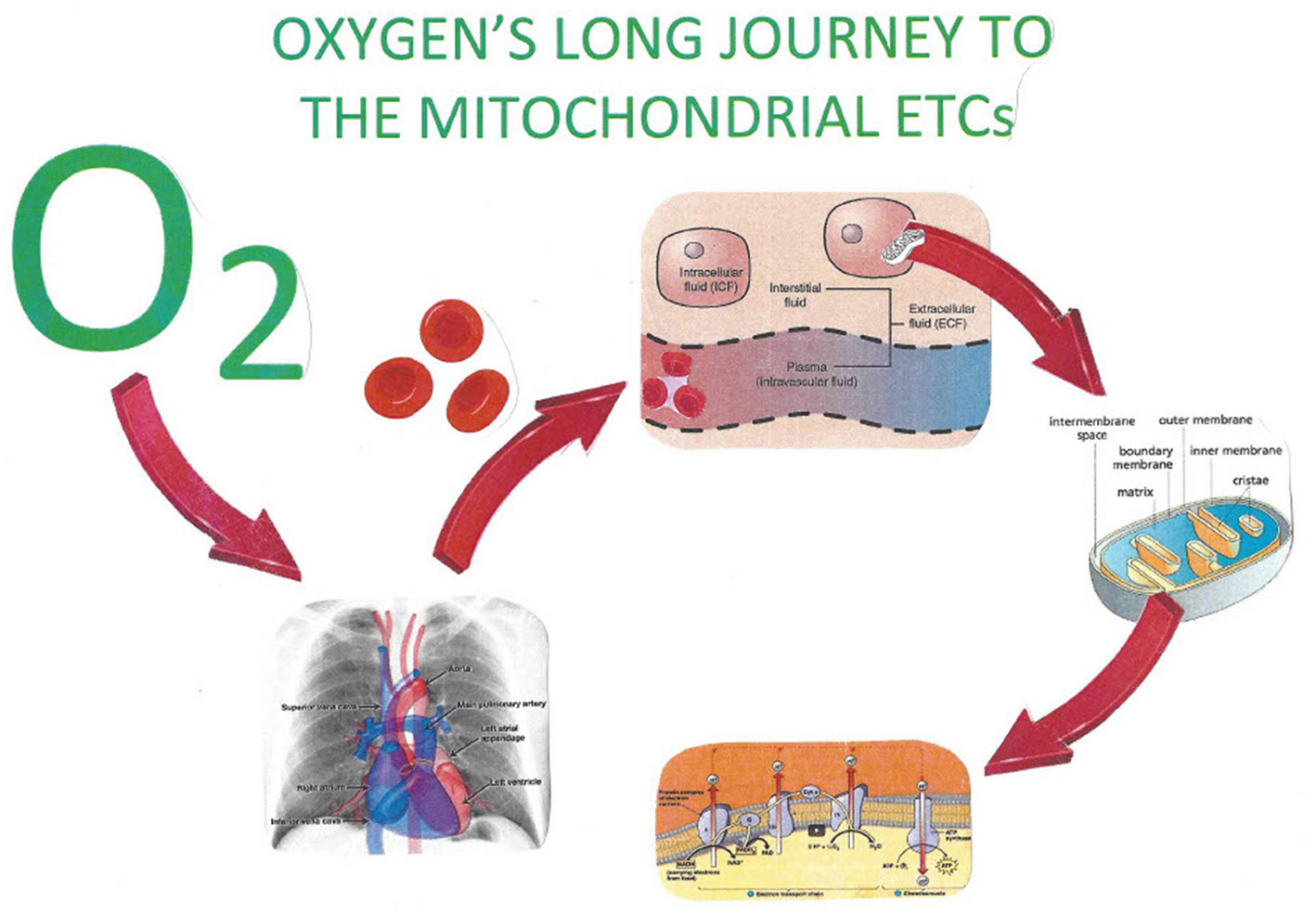
The increased ability of hyperbaric oxygen allows for an increased quantity of dissolved oxygen in body fluids. The facilitated delivery of oxygen thereby to the IMS of mitochondria throughout the body provides for necessary oxidative phosphorylation. Oxygen, by attaching to the cytochrome 3 oxidase enzyme of the mitochondrial electron transport chain, produces the necessary supply of hydronium ions for ATP.

Plasma delivers the oxygen from the red blood cells to the endothelium, where it diffuses into the interstitial fluid, then diffuses through cellular membranes into the cytosol, and finally passes into the intermembrane space (IMS) of mitochondria. HBO administered at three atmospheres of pressure (SEFIO_2_ 300%) allows 6.6 volume% of oxygen to be dissolved in plasma. It is this concentration that begins its journey by diffusion through the capillary endothelium, ultimately filling the IMS of mitochondria minimally with the 1.5–2.0 mmHg of dissolved oxygen requisite for the electron transport chain along with ATP synthase to produce ATP (107). Figures 3, 4 demonstrate this process.

**Figure 5:**
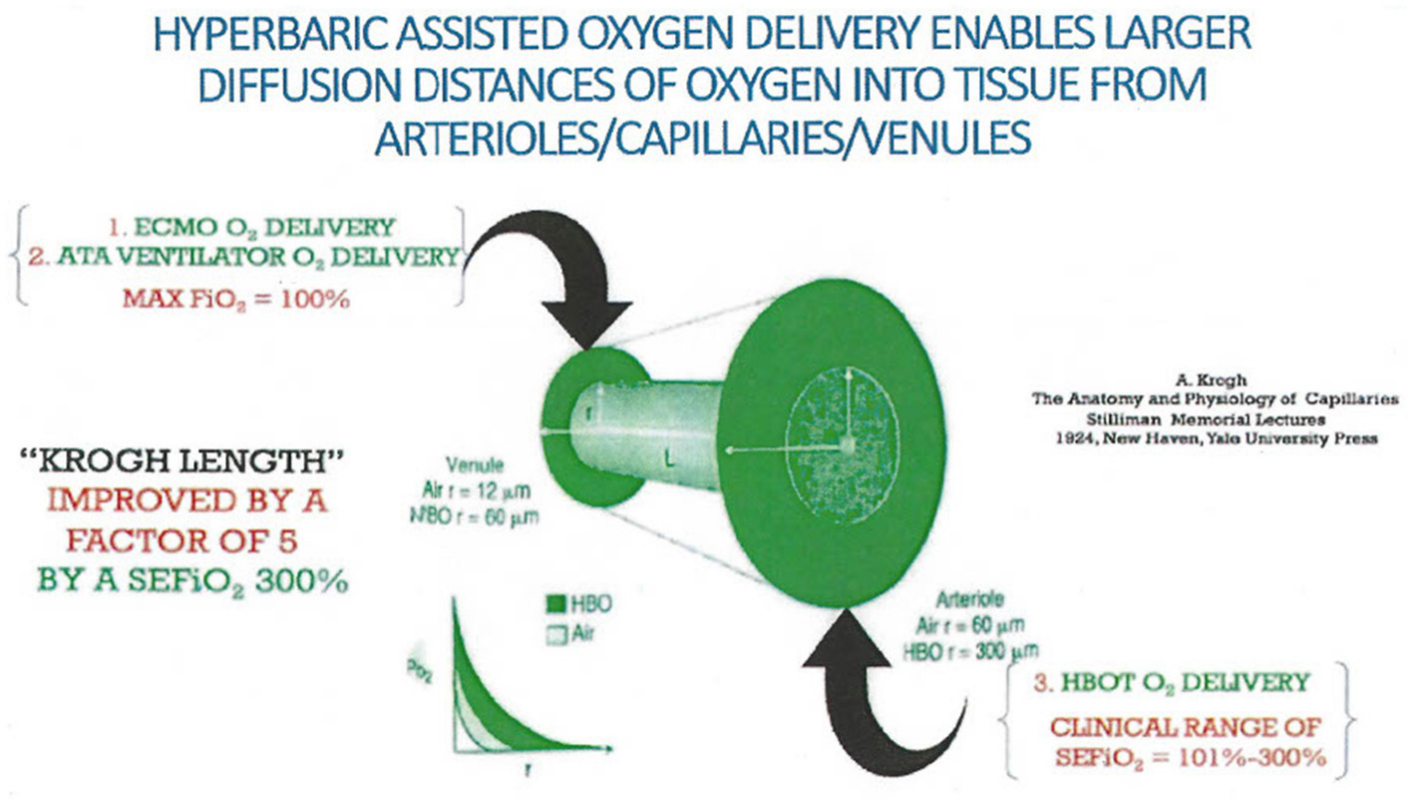
Krogh calculated the diffusion distance of oxygen outside of capillaries into tissue when a human breathed normobaric air. Extrapolation incorporating three atmospheres of pressure provided by hyperbaric treatment would provide a fivefold improvement in diffusion distance (94, 110).

The increased diffusivity of oxygen in tissue afforded by hyperbaric pressure is important. Krogh has described the diffusion distance of oxygen from plasma through the capillary endothelium (95, 97). This has been further expounded upon to include the added effect of the diffusivity of oxygen in the hyperbaric environment (99) as demonstrated in Figure 5.

Tissue oxygen capacitance increases during and after an HBOT treatment. The oxygen that is onloaded into the tissue during HBOT is, in part, slowly off-gassed, much like an inert gas with tissue elimination half-lives supplemented by the additional elimination of oxygen by metabolic consumption (109). Furthermore, some oxygen is retained in tissue by attaching to cellular gas clathrates [i.e., neuroglobin (110), cytoglobin (111), and myoglobin (112)]. With serial HBOT treatment, tissue oxygen capacitance increases (113).

**Figure 6:**
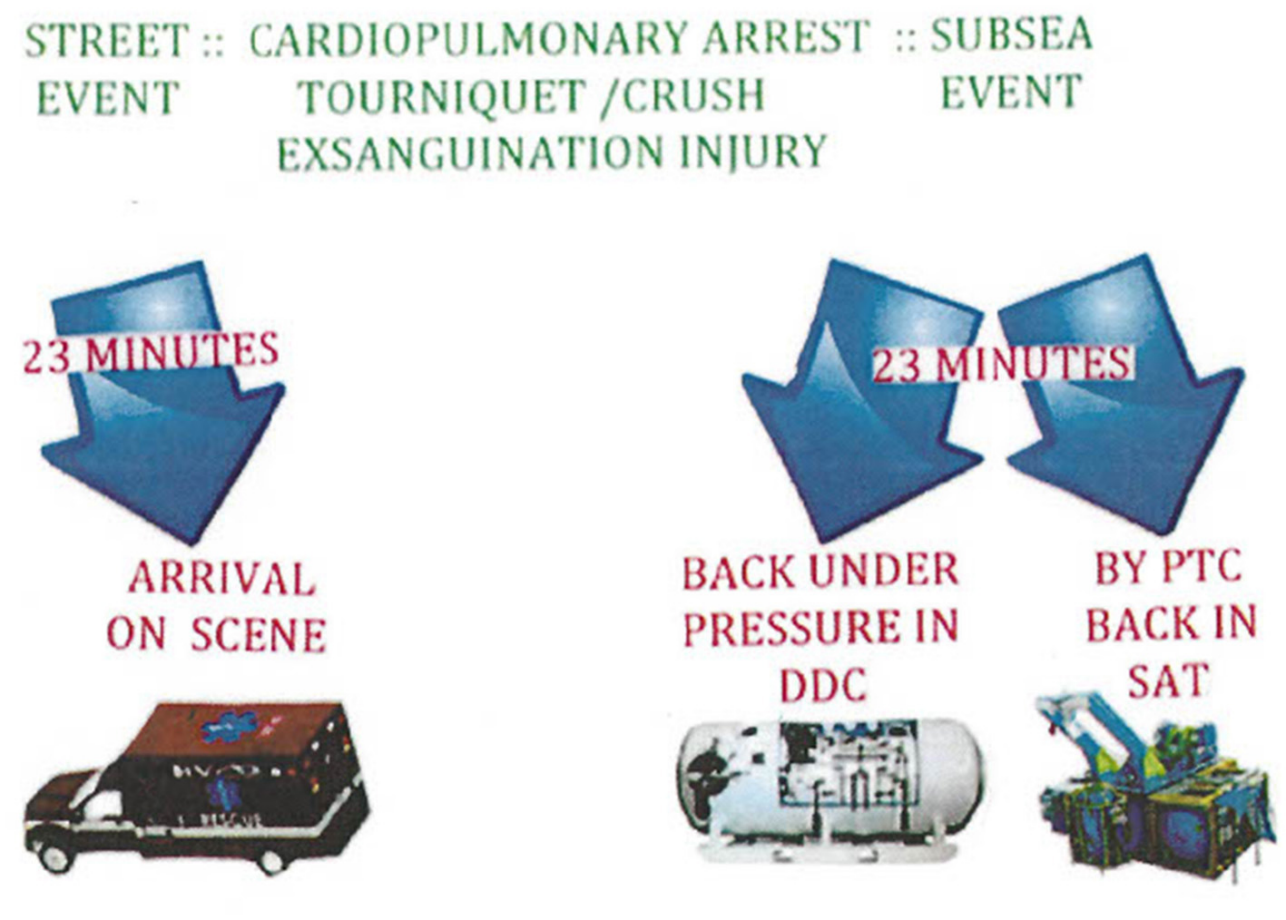
Having a hyperbaric ambulance to bring immediate hyperbaric oxygen treatment to severely anemic, injured, or ill patients in transit to a hospital emergency department increases the chance of having the same success rate as commercial diving operations, which require a hyperbaric chamber on site for accidents.

The red blood cell, as a biconcave disk, has a shape that maximizes its surface area. As a short-lived bag of hemoglobin, the mature red blood cell does not have mitochondria or a nucleus. An important mission of the red blood cell is to overcome the poor solubility of oxygen in plasma at one atmosphere in order to adequately get a supply of oxygen to mitochondria. The use of HBOT, especially in remote settings, has compelled some tertiary urban trauma medical staff to consider the development of a hyperbaric ambulance to mimic the success of the deck decompression chambers on operational sites to address injury of commercial divers (114). Figure 6 demonstrates this point.

A consideration of the potential toxic properties of a prolonged administration of O_2_ in almost all cases under normobaric, hyperbaric, or hypobaric exposures is a certainty (115–117). A judicious use of short-tie exposures of 60–90 min with intermittency of 5 min air breaks during administration and with gradual spreading of time intervals between treatments has thoroughly been documented to be safe, allowing the “hyperoxic–hypoxic paradox” prevail to the patient's benefit (118–120).

## Conclusion

Red blood cells play an important role in the chain of oxygen delivery to the mitochondrial IMS. Finally, in the IMS, oxygen attaches to cytochrome c oxidase in the electron transport chain of enzymes embedded in the inner IMS (121). Reacting with the oxygen and hydrogen ions, cytochrome c oxidase expels the by-product of water. The hydrogen ions in the IMS fall down the molecular shoot of the ATP synthase nanomachine, and by rotary catalysis, Pi ions join with ADP to form ATP. It is an evolutionary wonder that the red blood cell without mitochondria carts oxygen to mitochondria in all the body's cells to provide the energy for the homeostasis of life.

HBOT, if used promptly, can serve as a bridge therapy to alleviate illness and injury when transfusion of red blood cells is necessary, otherwise requiring massive transfusion protocols. In other instances, HBOT could address severely anemic patients burdened with complicating comorbidities that otherwise would preclude the desirability of transfusion of red blood cells in any amount or by transfusion altogether. Hypoxic stress simulates recovery, and recovery requires energy provided by ATP.

**Figure 7:**
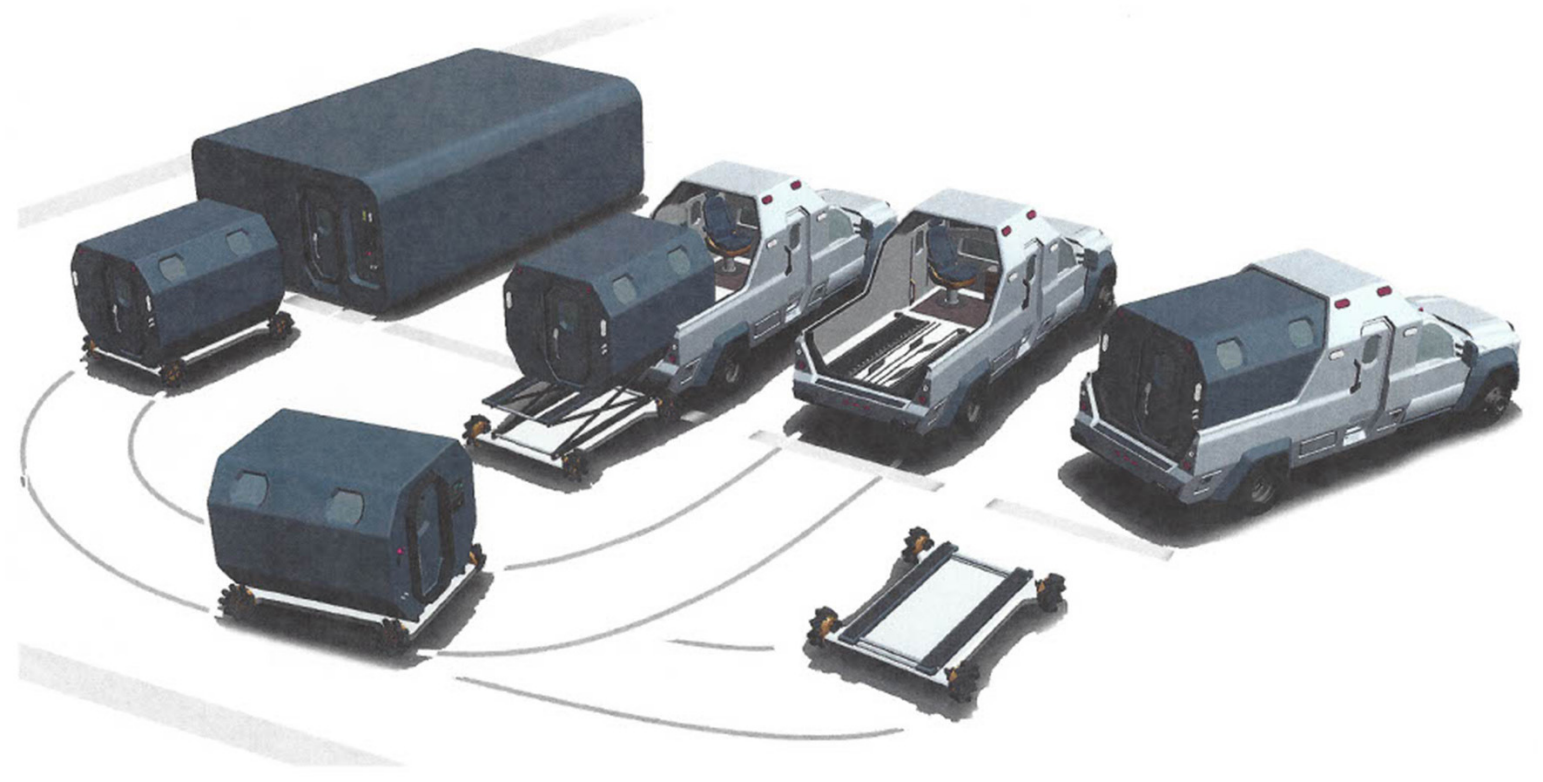
The best of all possibilities would be to have a hyperbaric ambulance system that deploys to the street to transport a severely anemic, ill, or injured patient under pressure with oxygen administration. Upon arrival at the hospital, the pressurized patient compartment would hydraulically depart the ambulance chaise and roll into the hospital emergency department to continue conventional normobaric resuscitation. The option could be for the detached patient compartment of the ambulance to mate with a hyperbaric intensive care multiplace chamber for continued hyperbaric resuscitation (113).

Emerging ways that HBOT can safely and quickly be available are currently in existence (122). Hyperbaric units can be parts of emergency departments (123), intensive care units (124), and ambulances (114). Figure 7 shows schematics for a designed hyperbaric ambulance. The cost of an HBOT treatment is equal to the cost of a unit of blood and its administration (4). HBOT does not need type and crossing, or IV access, as the systemic dose of oxygen is administered via breathing or through a patient ventilator.
